# Mitoxantrone‐Based Novel Conditioning Regimen Leads to Great Survival Benefit in Peripheral T‐Cell Lymphoma Compared to BEAM Regimen

**DOI:** 10.1002/cam4.70476

**Published:** 2024-12-09

**Authors:** Xinyu Zuo, Wensi Qian, Min Wu, Yanhui Xie, Jiexian Ma

**Affiliations:** ^1^ Department of Hematology, Huadong Hospital Fudan University Shanghai China

**Keywords:** autologous stem cell transplantation, carmustine, conditioning regimen, mitoxantrone, T‐cell lymphomas

## Abstract

**Background:**

Peripheral T‐cell lymphomas (PTCL) frequently result in relapsed or refractory diseases. Upfront autologous hematopoietic stem cell transplantation (ASCT) using the BEAM (carmustine, etoposide, cytarabine, and melphalan) regimen is recommended. However, relapses are common in PTCL, highlighting a critical need for improved survival outcomes in these patients.

**Objective:**

Anthracycline drugs are essential in treating PTCL. We compared the efficacy and tolerability of a high‐dose mitoxantrone‐based conditioning regimen [mitoxantrone, cyclophosphamide, and etoposide (MCE)] to the BEAM regimen in upfront ASCT for newly diagnosed PTCL patients who achieved complete or partial remission after induction therapy.

**Study Design:**

A retrospective study was conducted to analyze the treatment response, progression‐free survival (PFS), overall survival (OS), hematologic engraftment time, and adverse events of 64 patients between two regimens, who achieved complete or partial remission after induction chemotherapy. Twenty‐eight patients received the MCE regimen, while 36 patients were treated with the BEAM regimen.

**Results:**

There were no significant differences in clinical characteristics or the incidence of adverse events between the two groups. However, the median OS significantly favored the MCE group at 102.4 (95% CI, 87.0–117.8) months compared to 62.6 (95% CI, 50.8–74.5) months in the BEAM group (*p* = 0.023). Similarly, the median PFS was longer in the MCE group at 87.8 (95% CI, 65.8–109.8) months versus 42.5 (95% CI, 30.0–55.0) months in the BEAM group (*p* = 0.031).

**Conclusion:**

ASCT with the mitoxantrone‐based conditioning regimen is tolerable and appears to significantly improve the prognosis of PTCL patients, offering a promising alternative to the current standard of care.

## 1 Introduction

Peripheral T‐cell lymphoma (PTCL) is a rare subtype of non‐Hodgkin lymphoma (NHL), accounting for 5%–10% of cases in Western countries and approximately 25% in China, with more than 29 histologic subtypes [1]. The most common subtypes include PTCL, not otherwise specified (PTCL‐NOS), anaplastic large cell lymphoma (ALCL), and angioimmunoblastic T‐cell lymphoma (AITL) [2]. PTCL is associated with a significantly poorer prognosis compared to B‐cell lymphoma under current treatment regimens. The median overall survival (OS) for newly diagnosed patients is less than 30 months [3], while the median OS for relapsed patients is only 13.7 months [4, 5]. There is an urgent need to improve the prognosis of PTCL. The CHOP regimen (cyclophosphamide, doxorubicin, vincristine, and prednisolone) remains the most commonly used first‐line treatment for PTCL. The use of high‐dose chemotherapy followed by autologous hematopoietic stem cell transplantation (ASCT) during the first remission has been widely adopted by clinicians and is supported by clinical guidelines [6], although clinical outcomes are significantly influenced by the choice of conditioning regimen used in ASCT [7].

Conditioning regimens for ASCT in PTCL patients are similar to those used in NHL, including BEAM (carmustine, etoposide, cytarabine, and melphalan), BEAC (carmustine, etoposide, cytarabine, and cyclophosphamide), and CBV (cyclophosphamide, carmustine, and etoposide) [8, 9, 10]. BEAM is currently the most widely used conditioning regimen, with a 5‐year survival rate ranging from 58% to 67% [11, 12, 13, 14]. However, the relapse or disease progression rate within 3 years is as high as 34% after ASCT with the BEAM regimen [9, 15]. Additionally, the incidence of carmustine‐associated pulmonary toxicity ranges from 16% to 64%, which is linked to the development of idiopathic pneumonia syndrome. This adverse event significantly reduces progression‐free survival (PFS) and OS and increases the incidence of transplant‐related mortality [16, 17]. Consequently, modified conditioning regimens have been increasingly explored in transplant centers to enhance the efficacy, safety, and survival benefits of ASCT in PTCL patients [11].

Anthracycline‐based regimens remain the cornerstone of treatment for PTCLs [18]. Our previous research demonstrated that a novel conditioning regimen consisting of mitoxantrone, cyclophosphamide, and etoposide (termed MCE), followed by ASCT as upfront therapy, significantly prolonged PFS and OS in high‐risk diffuse large B‐cell lymphoma patients, and was well‐tolerated with complete hematological engraftment [19]. Based on these findings, we conducted this clinical study to compare the treatment response, tolerability, and safety of the MCE regimen with the BEAM regimen as conditioning regimens in newly diagnosed PTCL patients undergoing ASCT.

## 2 Materials and Methods

### 2.1 Patient Characteristics

A total of 64 PTCL patients were included in the study, comprising 45 patients with PTCL‐NOS, 12 patients with AITL, and 7 patients with anaplastic lymphoma kinase‐negative anaplastic large cell lymphoma (ALK‐ALCL). These patients underwent ASCT at Huadong Hospital from August 2013 to August 2023. Histopathological examinations of lymph nodes and bone marrow, along with immunohistochemistry, flow cytometry, and cytogenetics, were performed at the time of diagnosis. Key inclusion criteria were as follows: newly diagnosed PTCL patients who achieved complete remission (CR) or partial remission (PR) after induction therapy were enrolled to receive high‐dose chemotherapy plus autologous peripheral blood stem cell transplantation. Patients who did not achieve CR or PR after first‐line or second‐line induction therapy but refused allogeneic hematopoietic stem cell transplantation (allo‐HSCT) and were eligible and willing to receive ASCT were also included. Additional criteria included a Zubrod performance status score of < 3, left ventricular ejection fraction > 50%, absence of uncontrolled arrhythmia or unstable cardiac conditions, a corrected QT interval < 470 ms, no symptomatic pulmonary conditions with satisfactory pulmonary function tests, serum glutamic pyruvic transaminase levels < 4× the upper limit of normal, and serum bilirubin levels < 2× the upper limit of normal. Patients with concomitant malignancies (except nonmelanoma skin tumors and in situ cervical carcinoma), uncontrolled infections, pregnancy, or lactation were excluded. Written informed consent was obtained from all participants. Patients diagnosed with ALK‐positive ALCL, primary cutaneous lymphoma, lymphoblastic lymphoma, Sezary syndrome, or mycosis fungoides were excluded. Medical records were retrospectively reviewed and collected independently by two individuals to minimize bias. The study was approved by the Ethics Committee and Human Research Committee of Huadong Hospital.

### 2.2 Treatment Plan

After diagnosis, disease stage was evaluated according to the Ann Arbor staging system, involving a physical examination, blood and serum assays, and PET‐CT. Bone marrow aspirates and biopsies were obtained, and other staging procedures were performed. Standard variables of the International Prognostic Index (IPI) and other variables of known prognostic importance in these types of lymphomas were evaluated. CHOP regimen was the first‐line treatment for PTCL. Disease status was assessed every three chemotherapy cycles. If patients did not achieve PR, second‐line chemotherapy including ICE (etoposide, carboplatin, and ifosfamide) or CHOPE, or CHOP with CD30 antibody was adopted. Patients received chidamide maintenance during chemotherapy intervals. Patients who achieved CR or PR after six cycles of induction chemotherapy were enrolled for subsequent ASCT. All patients received chemotherapy (cyclophosphamide 30 mg/kg on Day 1 and etoposide 7.5 mg/kg on Days 1–2) combined with recombinant human granulocyte‐colony‐stimulating factor (rhG‐CSF) (5 μg/kg/day for 6 days) for mobilization.

### 2.3 Conditioning Regimen

Patients were divided into two groups (MCE group and BEAM group) based on the conditioning regimen they received. Patients diagnosed with PTCL between 2013 and 2023 received either the BEAM or MCE regimen according to their preference. Before making treatment decisions, patients are presented with data on the therapeutic outcomes of the MCE regimen in our center, as well as BEAM regimen for PTCL. The decision to administer either regimen was guided by patient consent following detailed discussions with their physicians. The MCE regimen consisted of high‐dose chemotherapy including mitoxantrone (60 mg/m^2^ on Day −1), cyclophosphamide (CTX, 60 mg/kg on Day −1), and etoposide (30 mg/kg on Day −1). The BEAM regimen included melphalan (140 mg/m^2^ on Day −1), cytarabine (Ara‐C, 400 mg/m^2^ from Days −5 to −2), etoposide (200 mg/m^2^ from Days −5 to −2), and carmustine (300 mg/m^2^ on Day −6). The total number of CD34+ cells collected was required to be more than 2 × 10^6^/kg, and the collected peripheral blood hematopoietic stem cells were frozen at −80°C in the BEAM group. In the MCE group, hematopoietic stem cells did not need to be frozen. The infusion of autologous peripheral blood progenitor cells was performed on Day 0. Patients received routine fungal prophylaxis, antiemetics, hydration, and urine alkalization. Low molecular weight heparin and alprostadil were used to prevent veno‐occlusive disease. If patients received high doses of cyclophosphamide, intravenous mesna was administered to prevent hemorrhagic cystitis. rhG‐CSF was administered starting on Day +1 after transplantation and continued until neutrophils ≥ 1.5 × 10^9^/L. Recombinant human thrombopoietin or recombinant human interleukin‐11 was given starting on Day +4 after transplantation and continued until platelet (PLT) levels reached ≥ 100 × 10^9^/L or for a maximum of 14 days. Blood counts were monitored, and PLT and red blood cell suspensions were administered as needed.

### 2.4 Response Assessment and Toxicity Criteria

Responses to treatment were graded according to the International Workshop on non‐Hodgkin's lymphomas criteria [20, 21]. CR was defined as the disappearance of all clinical, biological, and radiological evidence of lymphoma. PR was defined as a reduction of more than 50% in tumor burden. Progressive disease (PD) was defined as an increase of more than 25% in tumor mass or the appearance of new tumor masses. Cases that did not meet the criteria for CR, PR, or PD were classified as stable disease (SD). The following time‐to‐event endpoints were studied: OS, PFS, and time to hematologic engraftment. OS was calculated from the date of transplant to the date of death from any cause or the last follow‐up. PFS was defined as the time from stem cell infusion to disease progression, relapse, death from any cause, or the last follow‐up, whichever occurred first. If no event occurred, data were censored at the time of the last recorded patient contact. Neutrophil engraftment was defined as achieving a sustained absolute neutrophil count above 0.5 × 10^9^/L for three consecutive days. Platelet engraftment was defined as a platelet count exceeding 20 × 10^9^/L without the need for platelet transfusions for at least seven consecutive days. Toxicity assessment was performed according to the National Cancer Institute Criteria for Adverse Events, version 5.0, Cancer Therapy Evaluation Program (CTCAE) [22].

### 2.5 Statistical Methods

The database was closed for analysis as of June 2024. OS and PFS were calculated using the Kaplan–Meier estimate. Survival curves were generated using the Kaplan–Meier method and compared using the log‐rank test. Continuous data were reported as medians and ranges and analyzed using the Mann–Whitney *U*‐test. Categorical variables from patient characteristics were compared using chi‐squared tests, and, if necessary, Fisher's exact tests. To identify prognostic variables for OS and PFS, the Cox proportional hazards regression model was employed to evaluate the impact of multiple variables on the following clinical parameters: Age, sex, Ann Arbor stages, IPI, extra‐nodal involvement, B symptoms, serum LDH, response before ASCT, primary refractory status, engraftment, and treatment regimen. All statistical analyses were conducted using SPSS version 27.0 (SPSS, Chicago, IL) and GraphPad Prism version 9.0 (GraphPad Software Inc., La Jolla, CA, USA), with statistical significance defined as *p* < 0.05.

## 3 Results

### 3.1 Patient Characteristics

**FIGURE 1:**
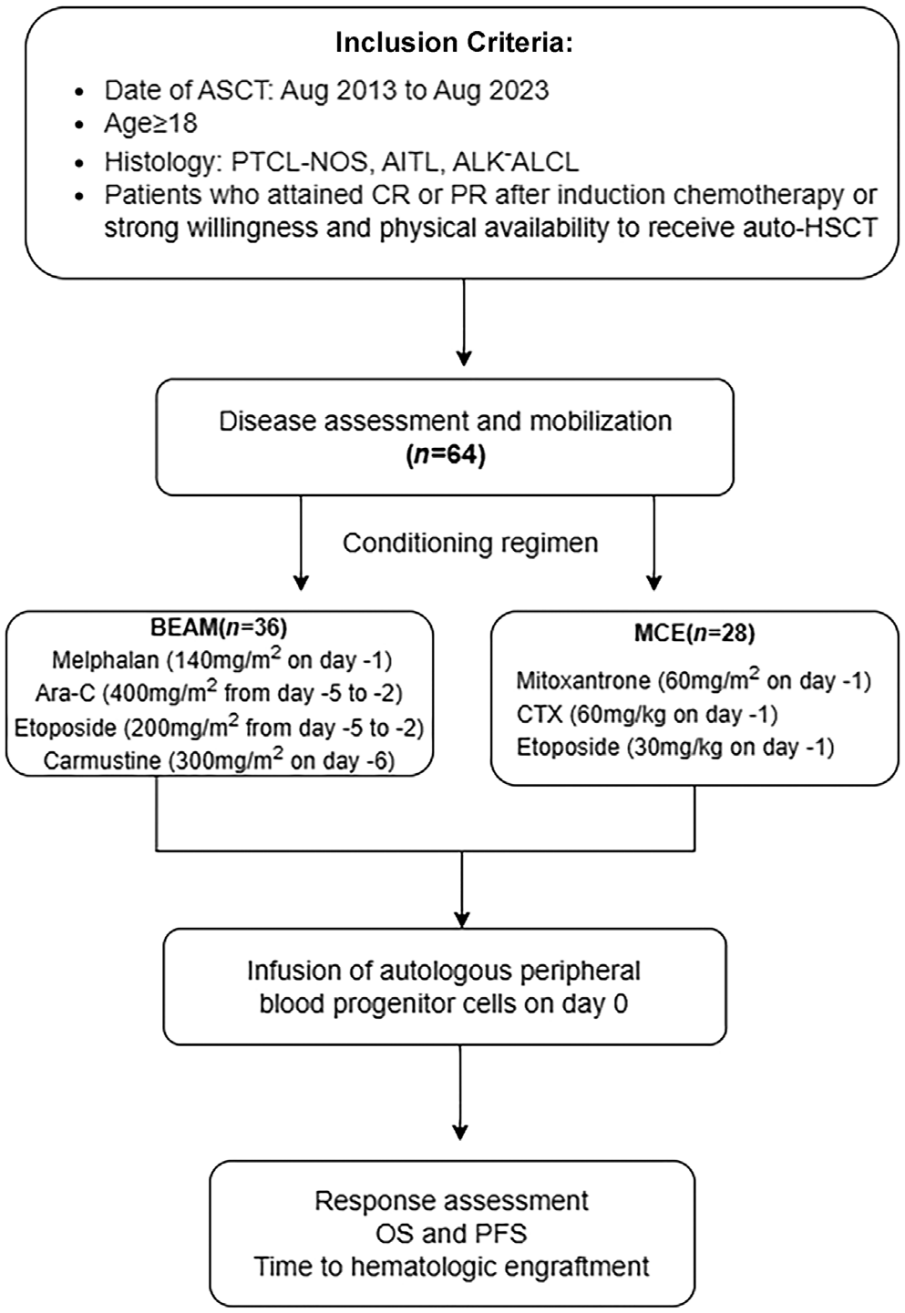
Flowchart of patient selection.

Table 1 presents the clinical characteristics of 64 patients with PTCL who underwent ASCT, showing no significant differences between the two groups. The flowchart is shown in Figure 1. A total of 55 PTCL patients who achieved complete or partial remission after induction chemotherapy, 2 PTCL patients who remained in SD and 7 patients presenting with PD were included in this study. Twenty‐eight patients were treated with the MCE regimen and 36 patients with the BEAM regimen. The clinical characteristics of patients in the MCE and BEAM groups were generally balanced across the cohort. The median age at diagnosis was 39 years in both groups (*p* = 0.704), and the disease subtypes at diagnosis were comparable between the groups (*p* = 0.108). The majority of patients were in Stage 3–4 (89.3% vs. 91.6%), with lactate dehydrogenase elevation observed in 82.1% and 88.9% of patients, and more than two‐thirds of patients had extranodal organ involvement. In the MCE group, more patients were refractory (39.29% vs. 22.22%) and had B symptoms (71.4% vs. 61.1%), though the differences were not statistically significant. Median chemotherapy cycles and responses before ASCT did not differ significantly between the two groups.

**TABLE 1:** Baseline clinical characteristics of patients receiving the two regimens.

Characteristics	MCE (*n* = 28)	BEAM (*n* = 36)	*p*
Age, years			0.704
Median (range)	39 (19–66)	39.5 (18–63)	
Gender, *n* (%)			0.033
Male	12 (42.9)	25 (69.4)	
Histological subtype, *n* (%)			0.108
PTCL‐NOS	16 (57.1)	29 (80.6)	
AITL	7 (25.0)	5 (13.9)	
ALCL ALK—	5 (17.9)	2 (5.6)	
Ann Arbor stage at diagnosis, *n* (%)			0.927
I	1 (3.6)	1 (2.8)	
II	2 (7.1)	2 (5.6)	
III	7 (25.0)	7 (19.4)	
IV	18 (64.3)	26 (72.2)	
IPI at diagnosis, *n* (%)			0.676
Low	5 (17.9)	7 (19.4)	
Low‐intermediate	7 (25.0)	9 (25.0)	
High‐intermediate	8 (28.6)	14 (38.9)	
High	8 (28.6)	6 (16.7)	
Extranodal involvement, *n* (%)	19 (67.9)	27 (75.0)	0.528
B symptoms, *n* (%)	20 (71.4)	22 (61.1)	0.389
Elevated LDH, *n* (%)	23 (82.1)	32 (88.9)	0.441
Primary refractory, *n* (%)	11 (39.29)	8 (22.22)	0.138
Cycles of chemo before ASCT			0.115
Median (range)	6 (3–13)	5 (3–12)	
Response before ASCT, *n* (%)			0.451
CR	13 (46.4)	21 (58.3)	
PR	13 (46.4)	13 (36.1)	
SD	1 (3.6)	0	
PD	1 (3.6)	2 (5.6)	
Allo‐HSCT, *n* (%)	2 (7.1)	6 (16.7)	0.448

Abbreviations: AITL, angioimmunoblastic T lymphoma; ALCL, anaplastic large cell lymphoma; ALK−, without anaplastic lymphoma kinase mutation; Allo‐HSCT, allogeneic hematopoietic stem cell transplantation; CR, complete remission; IPI, International Prognostic Index; LDH, lactate dehydrogenase; NOS, not otherwise specified; PD, progressive disease; PR, partial remission; PTCL, peripheral T‐cell lymphomas; SD, stable disease.

### 3.2 Clinical Outcomes and Overall Survival

**FIGURE 2:**
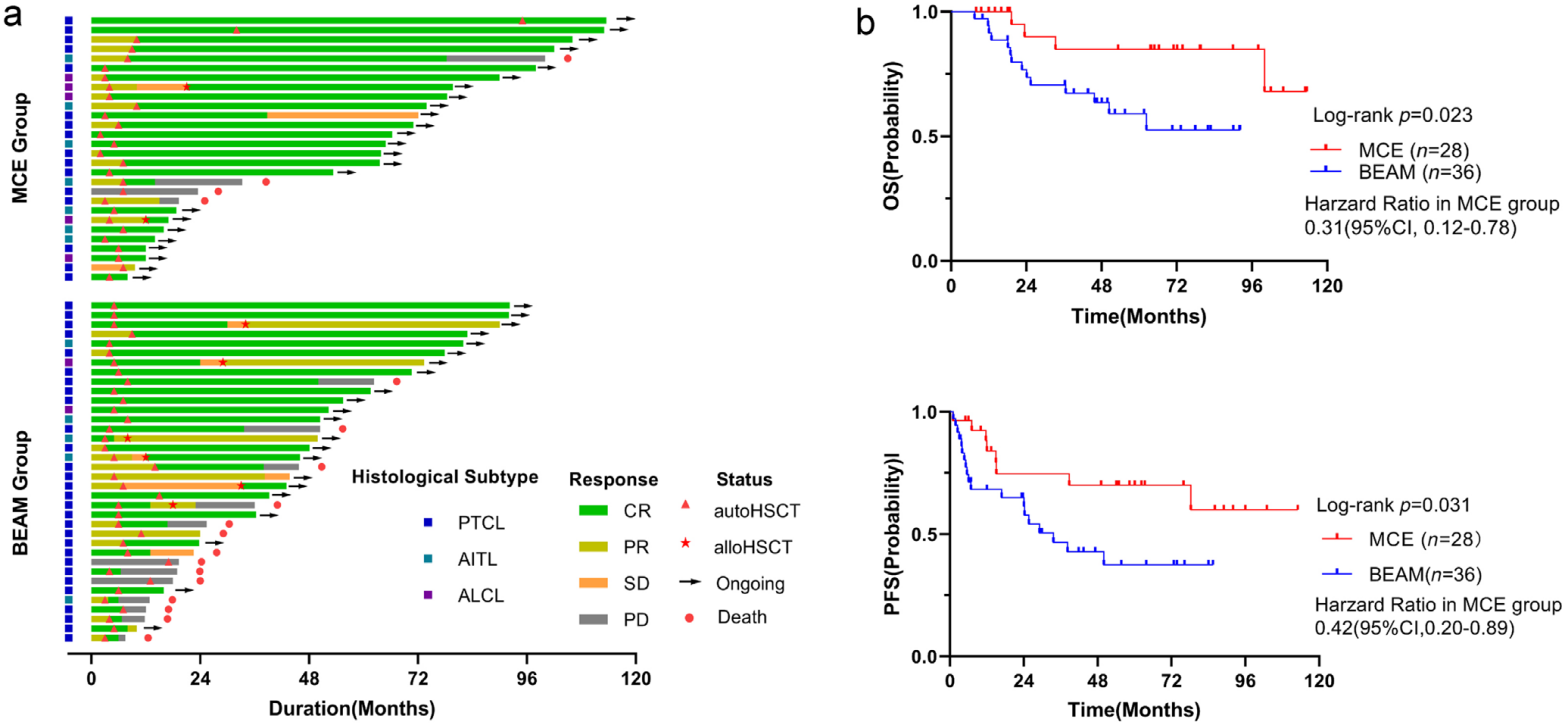
Analysis of the MCE regimen and BEAM regimen as conditioning regimens for ASCT in PTCL patients. (a) Swimmer plot of overall responses in PTCL patients. The onset and duration of response (CR, PR, SD, and PD) are indicated with specific symbols. Arrow: alive. Pentagram: allogeneic HSCT. Triangle: auto‐HSCT. (b) Overall survival and event‐free survival of the MCE regimen and BEAM regimen as conditioning regimens in PTCL patients. ASCT, autologous hematopoietic stem cell transplantation; BEAM, carmustine, etoposide, cytarabine, and melphalan; CR, complete remission; MCE, mitoxantrone, cyclophosphamide, and etoposide; PD, progressive disease; PR, partial remission; PTCL, peripheral T‐cell lymphomas; SD, stable disease.

The duration and depth of responses are presented in a swimmer plot (Figure 2a). In the MCE group, the median follow‐up time was 70.97 months (range: 8–120). In the BEAM group, the median follow‐up time was 55.5 months (range: 7.5–92.2), with no significant difference (*p* = 0.224). In the MCE group, 4 patients died, with a mean survival time of 102.4 months (95% CI: 87.0–117.8). In the BEAM group, 14 patients died, with a mean survival time of 62.7 months (95% CI: 50.8–74.5). There was a significant difference in OS time between the MCE and BEAM groups (*p* = 0.023, Figure 2b). A similar trend was observed in PFS (*p* = 0.038, Figure 2b), with a mean PFS of 87.8 months (95% CI: 65.8–109.8) in the MCE group and 42.5 months (95% CI: 30.0–55.0) in the BEAM group. The MCE group had a 3‐year and 5‐year OS of 85.0% and 85%, respectively, compared to 70.4% and 59.0% in the BEAM group. There was a significant difference in 5‐year OS (*p* = 0.023, Table 2) but not in 3‐year OS (*p* = 0.123, Table 2). The 3‐year relapse or progression rates were 21.4% in the MCE group and 47.2% in the BEAM group (*p* = 0.033, Table 2), indicating that the MCE regimen helped improve long‐term survival of PTCL patients.

**TABLE 2:** Outcome after ASCT.

	MCE (*n* = 28)	BEAM (*n* = 36)	*p*
Cumulative incidence of NRM, %	0 (0%)	1 (2.8%)	1
Cumulative incidence of relapse/progression, %			
At 1 year	14.3	30.6	0.149
At 3 years	21.4	47.2	0.033
PFS, %			
At 3 years	74.7	46.5	0.039
At 5 years	70.0	37.3	0.040
OS, %			
At 3 years	85.0	70.4	0.123
At 5 years	85.0	59.0	0.023

Abbreviation: NRM, non‐relapse mortality.

**FIGURE 3:**
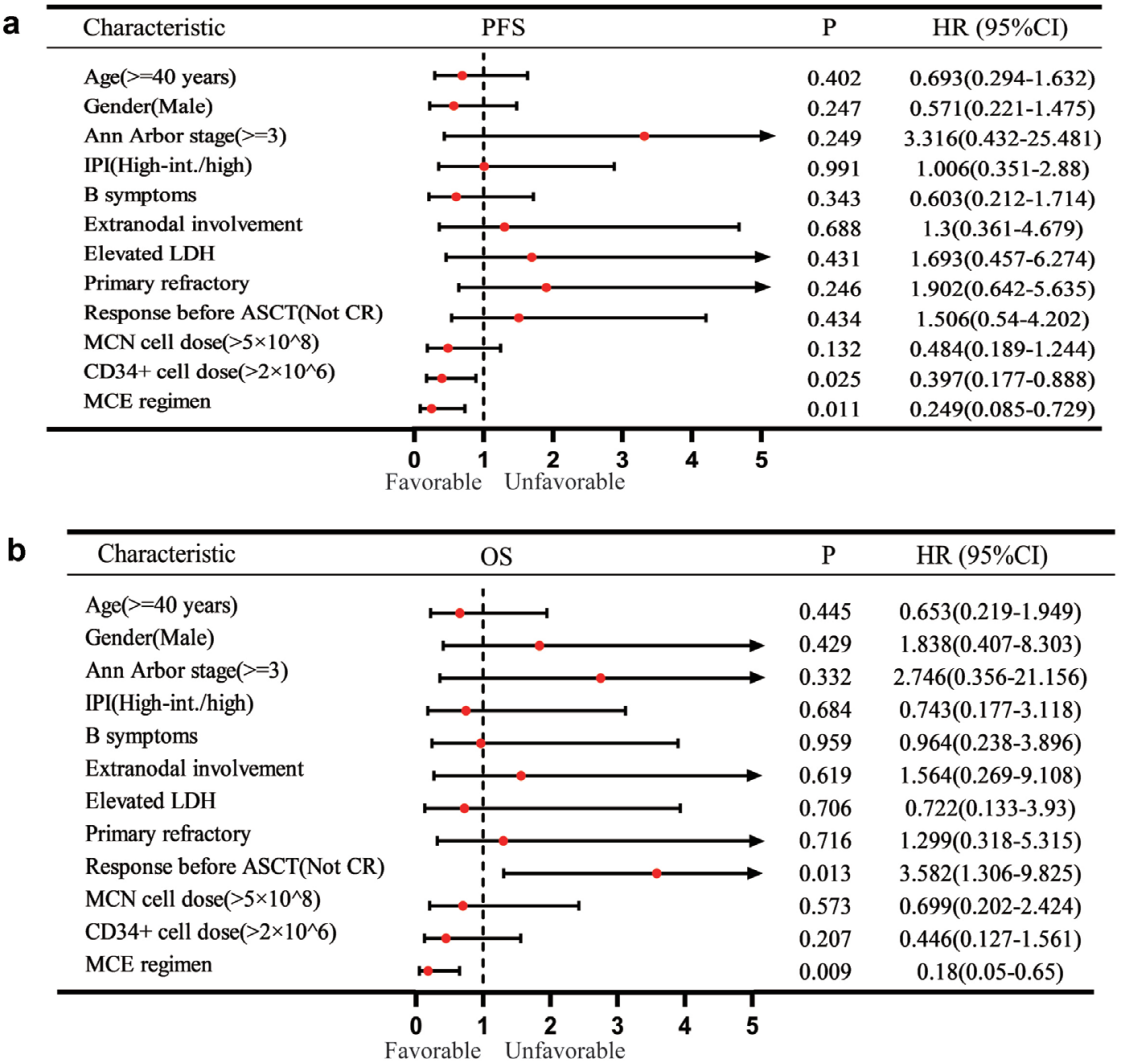
Forest plot of prognostic factors in PTCL patients. PTCL, peripheral T‐cell lymphomas.

To assess the impact of baseline characteristics on patient outcomes, we conducted multivariable analyses. Figure 3 compares PFS and OS in the entire cohort based on clinical features. The results show that failure to achieve CR after induction therapy was an adverse independent prognostic factor for OS (*p* = 0.013), and a CD34+ cell dose over 2 × 10^6^ was identified as a favorable prognostic factor for PFS (*p* = 0.025). Notably, the MCE regimen was a powerful favorable prognostic factor for both PFS and OS.

Due to the distinct prognostic and treatment response differences between patient groups, we conducted a subgroup analysis of high‐risk patients. The results of the subgroup analysis are shown in Table 3. High‐risk subgroups included those with an IPI score of 3–5, Ann Arbor stage III–IV, and primary refractory. In patients with primary refractory, the OS and PFS were significantly longer in MCE regimen with 98.5 months (95% CI: 76.0–120.95) compared to 36.9 months (95% CI: 14.57–59.13) (*p* = 0.018) and 74.2 months (95% CI: 43.43–104.98) versus 22.4 months (95% CI: 0–45.42) (*p* = 0.027). For patients receiving MCE regimen as first‐line therapy, PFS was significantly extended, with a mean of 71.6 months (95% CI: 54.34–88.90) compared to 46.93 months (95% CI: 33.20–60.66) for those with BEAM (*p* = 0.046).

**TABLE 3:** Outcome of subgroup.

	MCE	BEAM	*p*
PFS at 5 years, %			
Response before ASCT			
CR	77.1	47	0.046
PR	64.3	24.2	0.044
High risk			
IPI 3–5	60.8	18.9	0.047
Ann Arbor stage III–IV	65.4	37.4	0.218
Primary refractory	63.6	22.5	0.04
OS at 5 years, %			
Response before ASCT			
CR	100	76.5	0.685
PR	83.3	43.1	0.251
High risk			
IPI 3–5	90	62	0.043
Ann Arbor stage III–IV	82.4	57.8	0.045
Primary refractory	80	66.4	0.036
Cumulative incidence of relapse/progression at 3 years, %			
Response before ASCT			
CR	15.4	57.1	0.03
PR	16.7	33.3	0.409
High risk			
IPI 3–5	25	26.3	0.929
Ann Arbor stage III–IV	20	42.4	0.072
Primary refractory	45.5	50	0.845

Abbreviations: ASCT, autologous hematopoietic stem cell transplantation; BEAM, carmustine, etoposide, cytarabine, and melphalan; CR, complete remission; MCE, mitoxantrone, cyclophosphamide, and etoposide; PFS, progression‐free survival; PR, partial remission.

To assess whether the MCE regimen offers additional benefits for specific patient groups, we conducted a subgroup analysis of patients treated with the MCE regimen, with subgroup classifications outlined in Figure 3. Our analysis revealed patients acquired PR or CR did not have significant difference on PFS and OS. However, in the BEAM regimen group, OS was longer in patients who achieved CR with 74.68 months (95% CI: 61.59–87.76), compared to 44.59 months (95% CI: 28.62–60.55, *p* = 0.018), suggesting the potential of MCE regimen to eradicate residual tumors.

### 3.3 Engraftment and Toxicities

Tables 4 and 5 present the engraftment and toxicity data for the entire cohort. The median mononuclear cell (MCN) count in the MCE group was 5.95 × 10^8^/kg (range: 2.70–23.00). In the BEAM group, the median MCN count was 5.85 × 10^8^/kg (range: 3.00–10.50), with no statistically significant differences between the groups (*p* = 0.693). In the MCE and BEAM groups, the median doses of CD34+ cells were 2.25 × 10^6^/kg (range: 1.5–7.5) and 2.1 × 10^6^/kg (range: 1.3–10.70), respectively (*p* = 0.963). However, neutrophil engraftment occurred at a median time of 14 days (range: 10–40) in the MCE group, compared to 11.5 days (range: 9.8–35) in the BEAM group (*p* = 0.018). The median platelet engraftment time in the MCE group was 16 days (range: 10–50), which was significantly longer than in the BEAM group, which had a median of 13 days (range: 11–27) (*p* = 0.021). No instances of graft failure were observed in the study.

**TABLE 4:** Hematologic engraftment and response of the two regimens.

	MCE (*n* = 28)	BEAM (*n* = 36)	*p*
Days to neutrophil engraftment			0.018
Median (range)	14 (10–40)	11.5 (9.8–35)	
Days to platelet engraftment			0.021
Median (range)	16 (10–50)	13 (11–27)	
CD34+, ×10^6^/kg/day			0.963
Median (range)	2.25 (1.50–7.50)	2.1 (1.30–10.70)	
MCN, ×10^8^/kg/day			0.426
Median (range)	5.95 (2.70–23.00)	5.85 (3.00–10.50)	
Response after ASCT, *n* (%)			0.601
CR	23 (82.1%)	29 (80.6%)	
PR	4 (14.3%)	3 (8.3%)	
SD	0	1 (2.8%)	
PD	1 (3.6%)	3 (8.3%)	
Days of hospital, days			0.082
Median (range)	36 (23–61)	29.75 (21–54)	

Abbreviations: ASCT, autologous hematopoietic stem cell transplantation; MCN, mononuclear cell.

**TABLE 5:** Comparison of adverse effects associated with the two regimens.

	MCE (*n* = 28)	BEAM (*n* = 36)	*p*
Hematological			
Blood or bone marrow hypocellular, *n* (%)	23 (82.1)	31 (86.1)	0.664
Febrile neutropenia, *n* (%)	24 (85.7)	27 (75.0)	0.291
Infection			
Lung or upper respiratory infection, *n* (%)	1 (3.6)	3 (8.3)	0.625
Digestive system infection, *n* (%)	2 (7.1)	5 (13.9)	0.391
Mucosal infection, *n* (%)	3 (10.7)	3 (8.3)	1.000
Sepsis, *n* (%)	1 (3.6)	2 (5.6)	1.000
Non‐hematological			
Diarrhea, *n* (%)	2 (7.1)	4 (11.1)	0.688
Edema, *n* (%)	2 (7.1)	3 (8.3)	1.000
Vomiting, *n* (%)	13 (46.4)	15 (41.7)	0.703
Cardiac			
Tachycardia or prolonged QT interval, *n* (%)	2 (7.1)	0	0.188
Increased BNP, (*n* %)	3 (10.7)	3 (8.3)	0.768
Increased cTnT, (*n* %)	2 (7.1)	1 (2.7)	0.435
LVEF decreased (≤ 50%), (*n* %)	3 (10.7)	3 (8.3)	0.768
Heart failure, (*n* %)	3 (10.7)	3 (8.3)	1.000

Abbreviation: LVEF, left ventricular ejection fraction.

Most patients experienced Grade III–IV hematologic toxicity, with no significant difference observed between the MCE and BEAM groups (82.1% vs. 86.1%, *p* = 0.664). Febrile neutropenia was also common (85.7% vs. 75.0%, *p* = 0.291). Non‐hematologic toxicities, such as diarrhea and nausea/vomiting, were comparable between the MCE and BEAM groups. Cardiotoxicity and infection rates were also similar between the groups (*p* > 0.05, Table 4). Furthermore, over a 10‐year period, no patients were hospitalized due to adverse events related to cardiotoxicity.

### 3.4 Allo‐HSCT for Relapse/Progression Post‐ASCT

Among the relapsed and refractory PTCL patients after ASCT, allo‐HSCT was recommended as a salvage therapy for those eligible [19]. In the MCE group, 2 patients received allo‐HSCT and achieved CR; both were still alive at the time of analysis. In the BEAM group, 6 patients received allo‐HSCT; only 1 patient died after the procedure, while 4 patients did not achieve CR but were still alive at the time of analysis. Allo‐HSCT appeared to improve the survival of relapsed/refractory patients. Although more patients in the BEAM group underwent allo‐HSCT, the MCE group showed improved PFS and OS compared to the BEAM group.

## 4 Discussion

PTCL is a rare and heterogeneous group of clinically aggressive diseases associated with poor prognosis. Except for ALK+ALCL, the long‐term survival rate of other untreated PTCL types is less than 30% [23]. For patients with progressive or relapsed disease, long‐term disease control cannot be achieved solely through chemotherapy [24]. ASCT is recommended for PTCL patients after remission [13]. High‐dose chemotherapy combined with ASCT can improve survival in patients with chemotherapy‐sensitive diseases, both during upfront and salvage therapy [25].

The high‐dose chemotherapy regimen followed by reinfusion of hematopoietic stem cells is validated to minimize the tumor burden. As conditioning regimens are a crucial step for ASCT, several conditioning regimens are considered standard and routinely used for PTCL patients [26]. BEAM was the widely recommended conditioning regimen for PTCL. This study aimed to explore the long‐term survival outcomes, efficacy, and safety of a novel conditioning regimen in our medical center compared to the widely‐accepted BEAM regimen. The results showed that patients who received the MCE conditioning regimen had significantly prolonged 5‐year OS rates (85.0% vs. 59.0%, *p* = 0.023) and PFS (70.0% vs. 37.3%, *p* = 0.004) compared to the BEAM regimen. The MCE conditioning regimen improved the depth of remission in PTCL patients, reducing the 3‐year relapse rate from 47.2% to 21.4% compared to the BEAM regimen. Notably, the MCE regimen was a strong prognostic factor affecting OS and PFS in PTCL patients. Through subgroup analysis of patients with IPI scores of 3–5, refractory, and Ann Arbor stages III–IV, we found that the MCE regimen significantly prolonged survival. Notably, even patients PR benefitted from MCE treatment, further suggesting that this regimen not only effectively eliminates residual tumors but also serves as a powerful regimen for improving the prognosis of high‐risk patients. In the comparative analysis of treatment‐related complications between BEAM and MCE, no transplant‐related deaths were observed. The incidence of common adverse events, such as hematologic toxicity, nausea/vomiting, diarrhea, and infections, was not statistically different between the two groups. Importantly, no deaths related to cardiotoxicity were observed, and there were no significant differences in cardiac‐related adverse events between the MCE and BEAM groups. This suggests that the use of mitoxantrone and cyclophosphamide does not increase the risk of cardiotoxicity. However, a careful pre‐transplant cardiac assessment, especially for elderly patients, is still necessary before administering high doses of cyclophosphamide and mitoxantrone. Although the MCE regimen was associated with slower neutrophil and platelet engraftment compared to the BEAM regimen, which may be attributed to mitoxantrone inhibiting the migration and homing of CD34+ cells through the SDF‐1/CXCR4 signaling pathway. These factors were not independent prognostic factors in the multivariate analysis of PFS and OS, and the CD34+ cell counts showed no significant difference.

The treatment protocols used in this study differ from those commonly used in Western countries, based on clinical experience at our center and previously published studies [18, 27, 28]. We used consistent mobilization regimens in both the BEAM and MCE groups to ensure that this approach does not affect the generalizability of our results.

Although chidamide is not approved in Western countries, it has shown unique therapeutic benefits in in vitro experiments and clinical trials conducted in China [29, 30]. In our study, chidamide was administered consistently in both the BEAM and MCE groups, minimizing any potential bias.

There is limited data on mitoxantrone administration as a conditioning regimen in auto‐HSCT, yet more than 100 clinical cases in our medical centers (including DLBCL, PTCL, and NKT subtypes) have received the MCE regimen [18]. Our study indicates that the MCE conditioning regimen is effective and well‐tolerated for lymphoma patients undergoing ASCT. The MCE regimen provides a promising alternative to the traditional BEAM regimen for treating PTCL patients.

Our study has several limitations. First, the retrospective design inherently carries limitations. Second, the study's single‐center design and relatively small sample size constrain the generalizability of the findings. Third, due to low incidence of PTCL and limited number of patients tolerable to auto‐HSCT, this study spent a long time to enroll patients so as to induce some bias. Therefore, a multicenter, randomized controlled trial is necessary to provide a more reliable assessment of the comparative efficacy between the two treatment regimens.

## Author Contributions

J.M. and Y.X. designed the research study, oversaw the research, and critically reviewed the manuscript. W.Q. and M.W. collected patients' data. X.Z. managed the database, analyzed the data, and wrote the first draft of the paper. All authors revised the manuscript and approved the final version.

## Ethics Statement

The Huadong Hospital's institutional ethics committee approved the study. All procedures involving human subjects complied with the ethical standards of the institutional research committee and the Helsinki declaration, following guidelines approved by the Institutional Review Board (IRB) at Huadong Hospital.

## Conflicts of Interest

The authors declare no conflicts of interest.

## Data Availability

Correspondence and requests for materials should be addressed to Jiexian Ma.
